# Supplementary material for: High throughput method of 16S rRNA gene sequencing library preparation for plant root microbial community profiling
Source: Sci Rep. 2022 Nov 11;12:19289. doi: 10.1038/s41598-022-23943-x (PMC9652414; doi:10.1038/s41598-022-23943-x)
Supplement: Supplementary file 3 — Supplementary Information 3. [file 41598_2022_23943_MOESM3_ESM.pdf]

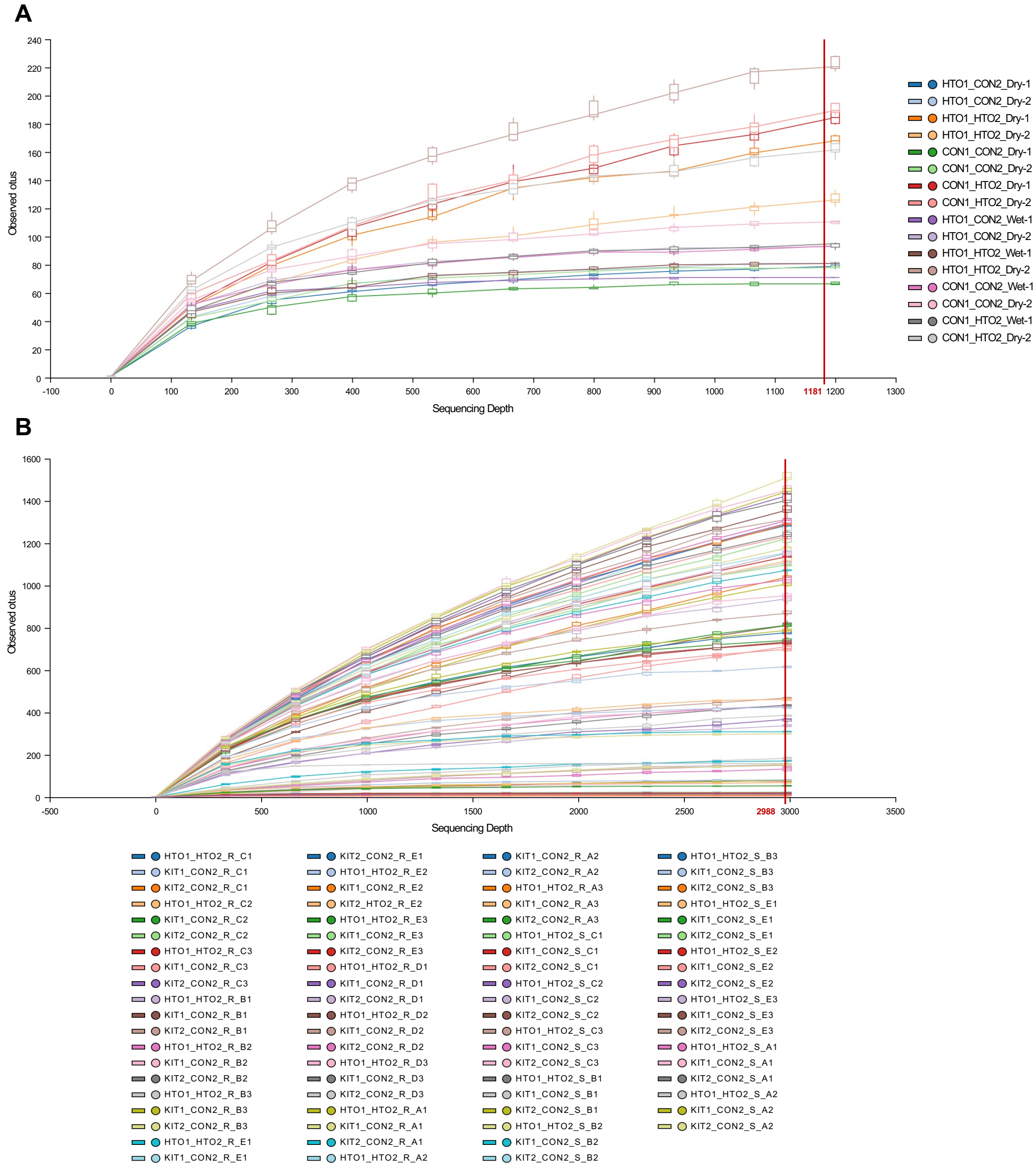

Figure S1. Rarefaction curve to determine the read number for the analysis

Rarefaction curves of ASVs across different samples for HTO1/2 vs. CON1/2 (**A**) and HTO1_HTO2 vs. KIT1/2_CON2 (**B**). Based on these results, we determined 1,181 and 2,988 reads as the depth parameter to rescue all samples and capture the differences in the ASVs among samples for the downstream analytical pipeline, respectively.

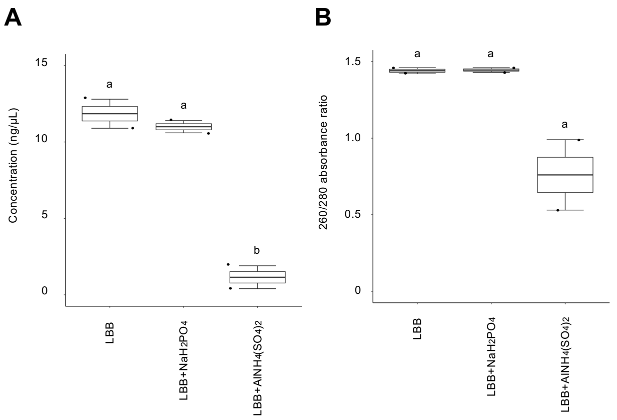

Figure S2. Comparison among different beads binding buffers

The yield (ng/100 mg sample tissue) (**A**) and quality (260/280 absorbance ratio) of nucleic acid (**B**) are shown for Lysate Binding Buffer (LBB) and its modified buffers combined with the AMPure XP bead DNA extraction methods. Different alphabet shows significant differences detected by Tukey test ($P < 0.05$). LBB and LBB + NaH_2PO_4 successfully extracted good quality DNA from soil samples. Based on this result, we used LBB for further experiments as HTO1.

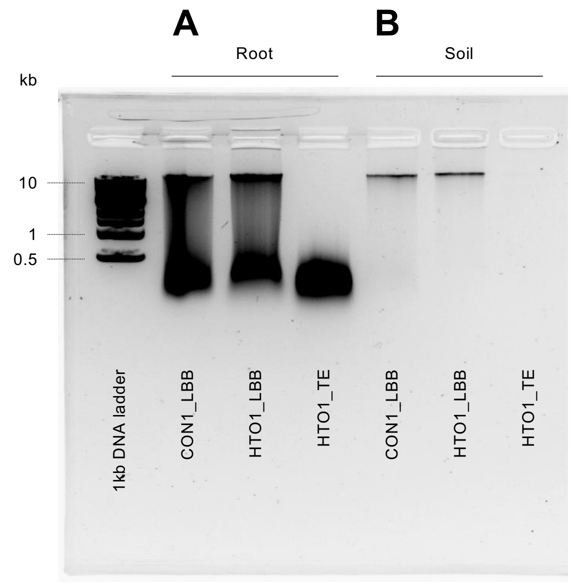

Figure S3. Agarose gel electrophoresis of genomic DNA extracted with different methods

The fragmentation status of the extracted DNA from the root (**A**) and soil samples (**B**) are shown.

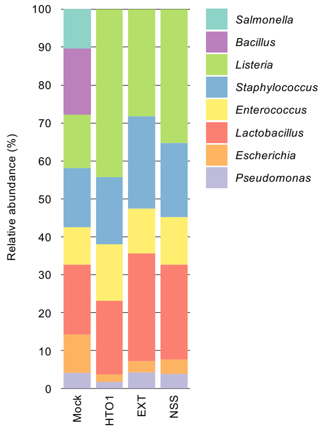

Figure S4. Mock microbial community experiment

The relative abundances of species in the mock microbial community standards in the HTO1_HTO2 method along with two different DNA extraction kits with combined with HTO2 method. Mock: Original composition of mock microbial community standards, EXT: Extrap Soil DNA Kit Plus Ver.2 (JBI, Cat. #212-006), NSS: NucleoSpin Soil (MACHEREY-NAGEL GmbH & Co. KG, Cat. #740780), which showed good performance in DNA extraction from soils (Miyaguchi, H. *et al.* Evaluation of the commercial kits for the extraction of extracellular DNA in soil. *Japanese Journal of Forensic Science and Technology* vol.24 63-72 (2018)). 6 mock species other than *Salmonella enterica* and *Bacillus subtilis* were detected in all methods with similar measurement bias, probably due to presence of the same species with mock species in original soil samples.

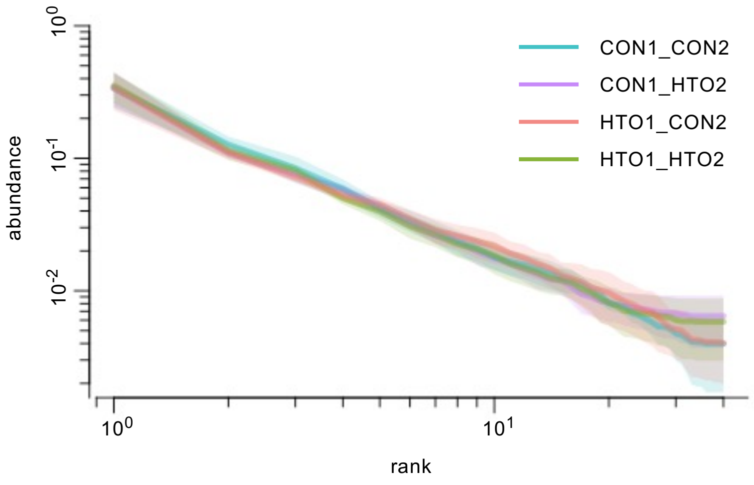

Figure S5. Normalized Rank Abundance Dominance (NRAD) plots obtained from different methods

Representative NRAD plots obtained from different methods are shown. The NRADs with stronger tails in the exonuclease method suggest that this method can detect minor bacteria.

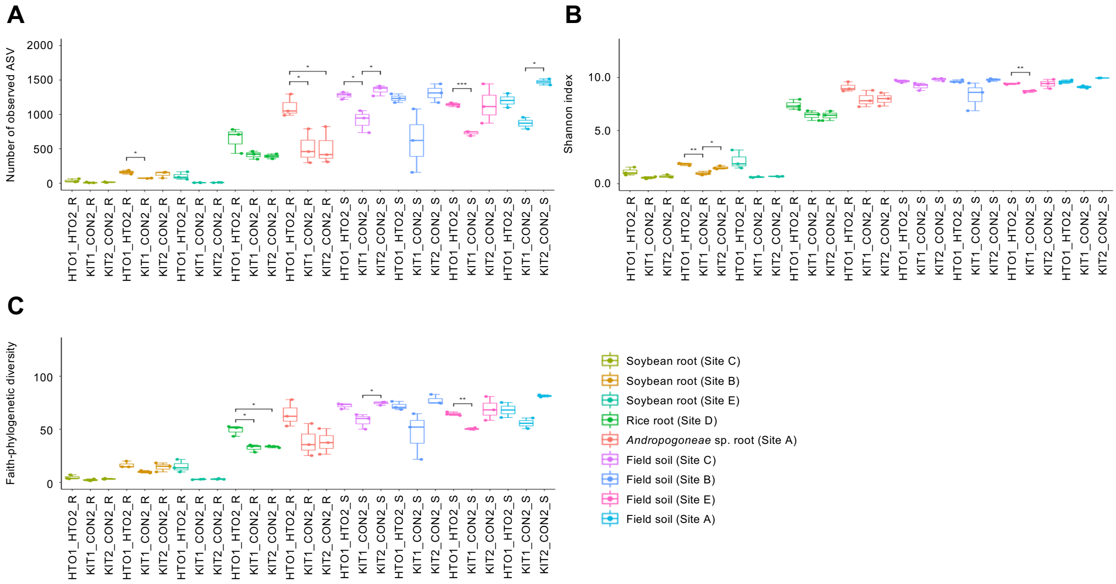

Figure S6. Alpha diversity metrics of plant root and soil microbial community diversity among new method and commonly used kits

Alpha diversity metrics of number of observed ASVs (A), Shannon diversity (B), and Faith phylogenetic diversity (C). Cultivated or growing naturally plant roots and various types of soils were sampled from different geographical sites: Site A, brown forest soil (36°N, 140°E); Site B, gray lowland soil (37°N, 140°E); Site C, gray lowland soil (43°N, 141°E); Site D, peat soil of rice paddy (43°N, 141°E); Site E, gley lowland soil (33°N, 130°E).

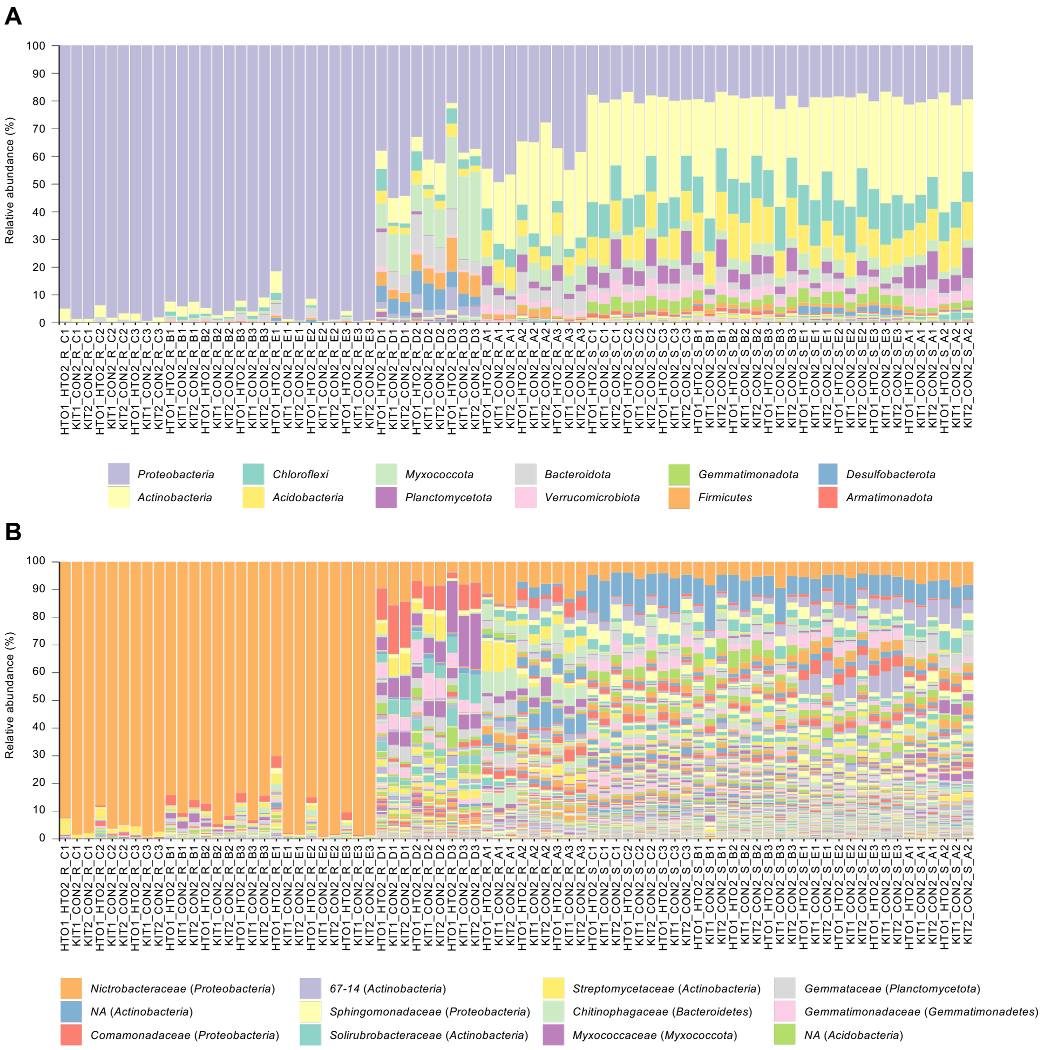

Figure S7. Taxonomic profile of plant root and soil microbiome among new method and commonly used kits

The relative abundances of major phyla (A) and family (B) are shown. Cultivated or growing naturally plant roots and various types of soils were sampled from different geographical sites: Site A, brown forest soil (36°N, 140°E); Site B, gray lowland soil (37°N, 140°E); Site C, gray lowland soil (43°N, 141°E); Site D, peat soil of rice paddy (43°N, 141°E); Site E, gley lowland soil (33°N, 130°E).
